# Time‐Varying Brain Functional Reconfiguration Patterns Associated With Fatigue in Multiple Sclerosis

**DOI:** 10.1002/hbm.70480

**Published:** 2026-03-08

**Authors:** Stefanie Hechenberger, Tommy A. A. Broeders, Marloes D. A. Bet, Birgit Helmlinger, Christian Tinauer, Stefan Ropele, Bettina Heschl, Sebastian Wurth, Anna Damulina, Michael Khalil, Menno M. Schoonheim, Christian Enzinger, Daniela Pinter

**Affiliations:** ^1^ Department of Neurology, Research Unit for Neuroplasticity and Repair Medical University of Graz Graz Austria; ^2^ Department of Neurology Medical University of Graz Graz Austria; ^3^ Clinical Department of Oncology, University Medical Center of Internal Medicine Medical University of Graz Graz Austria; ^4^ Amsterdam UMC, Vrije Universiteit Amsterdam, Anatomy and Neurosciences Amsterdam Neuroscience, MS Center Amsterdam Amsterdam the Netherlands; ^5^ Division of Neuroradiology and Interventional Radiology, Department of Radiology Medical University of Graz Graz Austria

**Keywords:** brain networks, fatigue, multiple sclerosis, time‐varying reconfigurations

## Abstract

Fatigue affects between 36.5% and 78% of people with multiple sclerosis (pwMS) and significantly impairs daily life. The neurobiological mechanisms underlying fatigue remain poorly understood and information about the time‐varying communication between brain regions and the networks they form may offer new insights into the complex pathology of MS‐related fatigue. Brain regions continuously reconfigure how they communicate within distinct networks (i.e., time‐varying reconfigurations) and aberrant time‐varying reconfigurations may contribute to the perception of fatigue in pwMS. This study aimed to explore if and how time‐varying reconfigurations are associated with fatigue in pwMS. In this cross‐sectional study, 155 pwMS (62% female; age = 39 ± 10 years; disease duration = 10 ± 8 years; median EDSS = 1.0 ± 2.0) and 48 healthy controls (HC) (71% female; age = 33 ± 10 years) underwent clinical, neuropsychological, and (resting‐state functional) MRI assessments. Fatigue was evaluated with the “Fatigue Scale for Motor and Cognitive Function”, comprising total, motor, and cognitive fatigue scores. Time‐varying connectivity was derived using a sliding‐window approach, with data‐driven assignment of brain regions to one of eight resting‐state networks for each window. Promiscuity (dispersion of reconfigurations), flexibility (frequency of reconfigurations), cohesion (joint reconfigurations), and disjointedness (independent reconfigurations) described the time‐varying reconfigurations of the whole brain and its networks. Among pwMS, 57% reported experiencing at least mild total fatigue (motor: 60%, cognitive: 57%). Higher total fatigue was correlated with greater global promiscuity (*r* = 0.21, *p* = 0.032) and disjointedness (*r* = 0.24, *p* = 0.008). Similarly, higher motor fatigue was associated with greater global promiscuity (*r* = 0.25, *p* = 0.008), flexibility (*r* = 0.21, *p* = 0.032), and disjointedness (*r* = 0.28, *p* < 0.001). The associations with disjointedness remained significant even after controlling for demographics, clinical measures, and structural brain damage, such as lesion load and atrophy (total fatigue: adj.*R* ^2^ = 0.23, *β* = 0.17, *p* = 0.033; motor fatigue: adj.*R* ^2^ = 0.38, *β* = 0.16, *p* = 0.026). Network‐level analyses in pwMS revealed that higher total (*r* = 0.25, *p* = 0.016) and motor (*r* = 0.25, *p* = 0.016) fatigue were associated with greater limbic network promiscuity. No significant correlations were found for cognitive fatigue in pwMS, or for total, motor, and cognitive fatigue in HC (all *p* > 0.05). Elevated levels of fatigue, particularly motor fatigue, in pwMS were linked to more unstable network reconfigurations, particularly of regions in the limbic network, possibly reflecting dysfunctional reward processing. More frequent dispersion requires more energy and may therefore contribute to increased fatigue.

**Trial Registration:**

This project was pre‐registered at ClinicalTrials.gov (registration number: NCT04892134)

## Graphical abstract

Fatigue in people with multiple sclerosis has been associated with more unstable and dispersed patterns of brain network reconfiguration, particularly within the limbic network. These findings suggest that inefficient and energetically demanding time‐varying communication among brain regions may contribute to the subjective experience of fatigue.

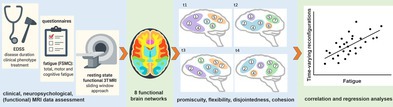

## 1 Introduction

Fatigue is one of the most common symptoms in persons with multiple sclerosis (pwMS), affecting between 36.5% and 78% and having a major impact on quality of life (Oliva Ramirez et al. 2021; David Ruban et al. 2021). MS‐related fatigue is defined as a subjective lack of physical and/or mental energy that is perceived by the individual with MS or caregiver to interfere with usual and desired activities (Penner and Paul 2017; Ayache and Chalah 2017). It can be subdivided into *motor fatigue*, involving reduced physical stamina, speed, and coordination, and *cognitive fatigue*, characterized by mental exhaustion and impaired concentration, attention, or memory (Mills 2012). This distinction facilitates investigation of underlying mechanisms and supports the development of targeted interventions (Penner et al. 2009).

Despite its clinical significance, the neurobiological basis of fatigue is poorly understood (Patejdl et al. 2016). Several studies exploring brain structure and function in MS have reported heterogeneous results highlighting that pwMS with fatigue showed more structural and functional alterations compared with pwMS without fatigue (Barbi et al. 2022). The most often reported structural finding associated with fatigue in MS is thalamic, striatal, and cortical (frontal, temporal, parietal) atrophy (Palotai and Guttmann 2020).

These findings underscore that fatigue in MS likely involves a range of distributed cortical and deep grey matter regions (Bertoli and Tecchio 2020). Studies using functional magnetic resonance imaging (fMRI) have been crucial to show how the communication between these brain regions (i.e., functional connectivity) may be altered in fatigued pwMS (Barbi et al. 2022). Accordingly, functional connectivity changes were reported in the prefrontal cortex, basal ganglia, and the limbic system, which are involved in reward processes (Heitmann et al. 2022; Romanello et al. 2022; Kampaite et al. 2024). In addition, to understand fatigue in pwMS we need to go beyond investigating regions in isolation and consider their embedding in functional brain networks (e.g., sensorimotor network [SMN], default mode network [DMN]) (Bertoli and Tecchio 2020; Bisecco et al. 2017; Cruz Gómez et al. 2013).

Building on this network‐level perspective, earlier MRI studies indicate that motor and cognitive fatigue in MS involve partly distinct neural mechanisms (Arm et al. 2019). Specifically, motor fatigue in pwMS is characterized by altered activation and functional connectivity in motor‐related regions such as the primary motor cortex, supplementary motor area, and cerebellum, as well as disrupted cortico‐striatal‐thalamic loops, potentially reflecting compensatory efforts associated with physical performance (Arm et al. 2019). In contrast, cognitive fatigue in pwMS has mainly been linked to altered activation and functional connectivity in executive and attentional networks, including the prefrontal cortex, anterior cingulate cortex, and fronto‐thalamic circuits, reflecting inefficient recruitment during sustained mental effort (Arm et al. 2019).

While these studies have provided valuable insights into the neural correlates of fatigue, most of them relied on static measures of functional connectivity (i.e., time‐averaged connectivity over the entire duration of a fMRI scan). However, functional connections are far from static and can change over time, even within a single resting‐state fMRI scan (Broeders et al. 2022; Broeders, Linsen, et al. 2024). Importantly, these temporal changes in functional connectivity—hereafter referred to as time‐varying functional connectivity—were often found to be behaviorally relevant by themselves (Preti et al. 2017). Two previous studies reported alterations in time‐varying functional connectivity associated with fatigue in pwMS (Romanello et al. 2022; Tijhuis et al. 2021). Tijhuis et al. (2021) found lower basal ganglia‐DMN time‐varying functional connectivity associated with higher fatigue values in pwMS (Tijhuis et al. 2021). Romanello et al. (2022) showed that higher fatigue severity scores were associated with a decreased overall connectivity between the basal ganglia and the fronto‐parietal control network (Romanello et al. 2022). These findings highlight the relevance of the time‐varying framework for understanding fatigue in pwMS, while studies specifically on cognitive and motor fatigue are still missing.

In addition, methodological advances have enabled a detailed characterization of the time‐varying reconfiguration of specific networks, rather than focusing on the connectivity of the entire brain; in other words: examining how a region is continuously and dynamically reconfigured across networks and whether brain regions are reconfigured in unison or individually (Telesford et al. 2016), which showed unique and complementary insights into the neural underpinnings of several behavioral outcomes in healthy individuals (Bassett et al. 2011; Braun et al. 2015). This method has already been applied to pwMS as well, showing that more intense network reconfigurations were associated with more impaired cognition (Broeders et al. 2022) and clinical disability (von Schwanenflug et al. 2023) in MS. Increased time‐varying reconfigurations may require more effort and put more burden on the network (Broeders, Van Dam, et al. 2024), thereby potentially playing an important role in the development of fatigue in MS. However, until now the association between these time‐varying brain reconfiguration patterns and (motor/cognitive) fatigue has not been studied for pwMS.

**FIGURE 1:**
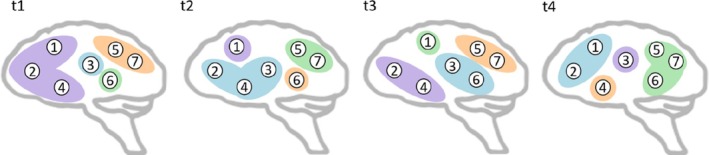
Simplified representation of time‐varying network reconfiguration (promiscuity, flexibility, cohesion, disjointedness). Figure 1 (adapted from Broeders, Van Dam, et al. 2024) illustrates a simplified representation of time‐varying network reconfiguration. **Promiscuity** quantifies how many networks a brain region is assigned to relative to the total number of networks (e.g., region 1 belongs to 3 of 4 networks: violet, green, and blue). **Flexibility** captures how often a region changes its network assignment over time (e.g., region 2 switches 3 times out of 3, while region 3 switches once out of 3). **Cohesion** measures how many reconfigurations occur jointly with another region (e.g., regions 2 and 4 switch together from t1 to t2 and from t2 to t3; region 5 and region 7 switch together from t1 to t2, from t2 to t3, and from t3 to t4). **Disjointedness** reflects how many reconfigurations occur independently (in this example, most switches are independent; e.g., region 1 independently switches from t2 to t3 and from t3 to t4).

To address this gap, we, therefore, investigated time‐varying global and network reconfigurations by calculating graph‐theoretical time‐varying reconfiguration measures such as promiscuity, flexibility, cohesion, and disjointedness (Figure 1). Using these time‐varying parameters, associations with (motor/cognitive) fatigue in pwMS and healthy subjects were examined. As suggested in recent literature (Tijhuis et al. 2021), using this more advanced time‐varying approach might help to gain new insights into the complex pathology of MS‐related fatigue.

## 2 Methods

### 2.1 Participants

For this study, 155 pwMS and 48 healthy controls (HC) were recruited from the outpatient clinic of the Medical University of Graz, Department of Neurology. MRI, neuropsychological, and clinical data were assessed between 2021 and 2023. All participants underwent MRI and neuropsychological assessment on the same day, except for 11 participants in whom the interval between assessments ranged from 1 to 24 days.

Detailed information about demographics, clinical, neuropsychological, and MRI data is presented in table 1.

All pwMS were diagnosed (Polman et al. 2011; Thompson et al. 2018) with clinically isolated syndrome (CIS) or definite MS (relapse‐remitting (RRMS), secondary progressive (SPMS), or primary progressive (PPMS)), were relapse‐free without steroid treatment for at least 8 weeks before MRI and neuropsychological assessment, and had no history of current psychiatric and/or neurological disease besides MS.

This project was approved by the Ethics Committee of the Medical University of Graz (31–432 ex 18/191264–2019) and pre‐registered at ClinicalTrials.gov (registration number: NCT04892134). Written informed consent was obtained from all participants. The study was performed in accordance with the Declaration of Helsinki.

### 2.2 Neuropsychological Assessment

Fatigue was assessed using the “Fatigue Scale for Motor and Cognitive Functions” (FSMC), providing a score for total, motor, and cognitive fatigue (Penner et al. 2009). Participants were asked to answer 20 questions on a 5‐point Likert scale (total fatigue maximum score: 100; motor and cognitive fatigue maximum score: 50). Fatigue severity was classified using the recommended cut‐off values. For total fatigue, scores ≥ 43 were defined as mild fatigue, ≥ 53 as moderate fatigue, and ≥ 63 as severe fatigue. For motor and cognitive fatigue, scores ≥ 22 were classified as mild motor/cognitive fatigue, ≥ 27/28 as moderate motor/cognitive fatigue, and ≥ 32/34 as severe motor/cognitive fatigue (Penner et al. 2009).

To distinguish fatigue from anxiety and depression, we administered the Hospital Anxiety and Depression Scale (HADS) (Feinstein et al. 2014), with scores ≥ 11 signifying moderate anxiety/depression (clinically significant) (Zigmond and Snaith 1983).

### 2.3 Clinical Assessment

Specialized neurologists assessed the clinical phenotype, level of physical impairment (Expanded Disability Status Scale; EDSS) (Kurtzke 1983), and gathered information concerning disease modifying treatment (DMT) and disease duration (DD) in pwMS.

### 2.4 MRI‐Protocol

MRI of the brain was performed on a 3 T scanner (Siemens MAGNETOM 3 T Prisma Fit system) at the Department of Radiology, Medical University of Graz, Austria. To enable assessment of normalized cortical/subcortical brain volumes, high‐resolution 3D images were acquired using a T1‐weighted MPRAGE sequence with 1 mm isotropic resolution (repetition time (TR): 1900 ms, echo time (TE): 2.7 ms, inversion time (TI): 900 ms). A T2‐weighted 3D Fluid‐Attenuated Inversion Recovery (FLAIR) sequence with 1 mm isotropic resolution was used for the assessment of hyper‐intense T2 white matter lesion‐load (T2‐LL) in pwMS (TR: 5000 ms, TE: 393 ms, TI: 1800 ms). To explore time‐varying reconfigurations, a single shot multiband echo planar imaging sequence (resting‐state‐fMRI) with 2 mm isotropic resolution was used (TR: 1000 ms, TE: 35 ms, field of view 256 × 256 mm^2^, acquisition time 5.20 min, 2 mm isotropic resolution, 300 volumes, 52 slices). During the resting‐state‐fMRI sequence, the lights were turned off and participants were instructed to close their eyes and remain awake.

### 2.5 Structural MRI Analyses

To assess T2‐weighted hyper‐intense white matter lesion load (T2‐LL), we employed the Lesion Segmentation Toolbox on SPM12, using the automated lesion prediction algorithm (LPA) (Schmidt 2017) on FLAIR images. After segmentation, lesion masks were visually checked and manually corrected when necessary. A binary lesion mask (threshold = 0.25) was then generated with fslmaths (FSL, v6.04), and the volume of T2‐LL (in cm^3^) for each pwMS was extracted using fslstats (FSL) (Smith et al. 2004).

After lesion‐filling with the FSL lesion‐filling toolbox (Battaglini et al. 2012), brain volumes were quantified, and tissue type segmentation was conducted based on T1‐weighted MPRAGE images utilizing SIENAX (Smith et al. 2002), a component of the FMRIB Software Library (FSL) (Smith et al. 2004). The resulting total, cortical grey matter, white matter, and cerebrospinal fluid volumes were normalized for head size using the V‐scaling factor derived by SIENAX. Subcortical volumes were determined from T1‐weighted images using FSL FIRST (Patenaude et al. 2011).

### 2.6 Functional MRI Analyses—Preprocessing

In a first step, all images were visually inspected to check for scanner artifacts, excessive motion and other distortions. Thereafter, several steps of the preprocessing pipeline FEAT (FSL 6.0.4) (Woolrich et al. 2001) were applied: motion correction using MCFLIRT, brain extraction, spatial smoothing using a Gaussian kernel of 5 mm full‐width at half‐maximum, linear registration to the individual T1 image, non‐linear registration to the Montreal Neuroscience Institute (MNI) standard space, and motion artifact removal using ICA‐AROMA (Pruim et al. 2015). White matter and cerebrospinal fluid masks derived from SIENAX, excluding subcortical structures and ventricle volumes, were linearly transformed to functional data. The mean white matter and cerebrospinal fluid signals were then regressed out from the BOLD images. Finally, high‐pass temporal filtering (0.01 Hz) was applied. After all preprocessing steps, the images were visually inspected again to ensure that all steps had been performed correctly.

### 2.7 Functional MRI Analyses—Network Registration

**FIGURE 2:**
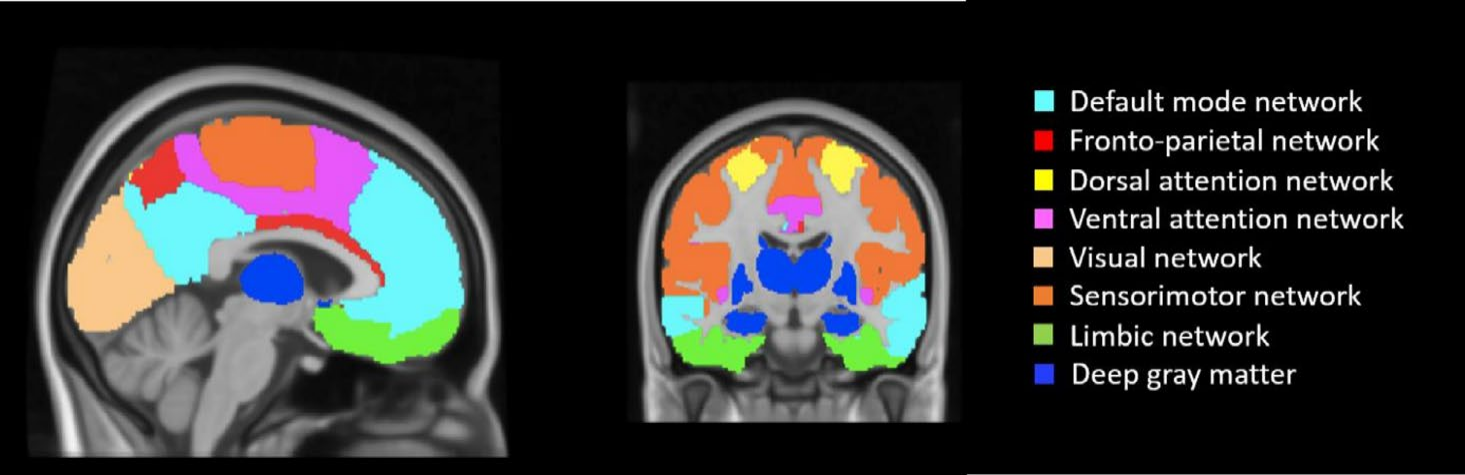
Illustration of the 8 resting‐state networks. Eight resting‐state networks are shown: The default mode (DMN, turquoise), fronto‐parietal (FPN, red), dorsal attention (DAN, yellow), ventral attention (VAN, pink), visual (VN, light orange), sensorimotor (SMN, dark orange), limbic (LN, green), and deep gray matter (DGM, blue) networks.

All 210 cortical regions from the Brainnetome atlas (Fan et al. 2016) were combined with 14 deep gray matter regions (nucleus accumbens, amygdala, nucleus caudatus, hippocampus, pallidum, putamen, thalamus; left and right hemisphere respectively) segmented using FIRST, which were transformed to standard space using inverted registration parameters of the 3DT1 scans. Voxels that represented white matter or cerebrospinal fluid (based on SIENAX tissue segmentation) were excluded. Twenty‐four regions that had < 30% voxel coverage in more than 10% of all participants were excluded from the entire analyses (Broeders et al. 2022). The remaining 200 regions for pwMS and HC were assigned to one of seven literature‐based defined resting‐state networks: DMN, fronto‐parietal network (FPN), dorsal attention network (DAN), ventral attention network (VAN), visual network (VN), SMN, and limbic network (LN) (Thomas Yeo et al. 2011). Furthermore, all 14 deep gray matter regions were grouped into one separate network (DGM), resulting in a total of eight networks (Figure 2).

### 2.8 Functional MRI Analyses—Community Detection and Time‐Varying Network Connectivity

Time‐varying connectivity was derived using a sliding‐window approach with a window‐size of 60s and a step‐size of 10s (yielding 25 windows), as these parameters have been suggested to capture the full range of time‐varying network reconfiguration (Preti et al. 2017). Windowed connectivity was calculated using absolute Fisher *r*‐to‐*z* transformed Pearson correlations. Community detection was done using a data‐driven, previously published approach (Broeders et al. 2022; Broeders, Linsen, et al. 2024). In brief, the initial phase involved the assignment of brain regions to literature‐based resting‐state networks. Subsequently, a cyclic process of network re‐evaluation ensued, wherein regions displaying the lowest assignment quality were systematically reallocated to alternative networks based on their maximal connectivity. This iterative refinement persisted until the selection of the same brain region occurred in consecutive iterations, indicating a point of saturation in assignment optimization. The used MATLAB (2020b) script is publicly available on GitHub (https://github.com/taabroeders/Recon_Dyn_MS/blob/main/CommunityDetection.m). As a result, each pwMS/HC contributed a 200 × 25 matrix (regions×windows), encapsulating the final network assignment across all regions within each temporal window (Broeders et al. 2022; Broeders, Linsen, et al. 2024).

Time‐varying reconfigurations of the whole brain (global) and of the eight networks (DMN, FPN, DAN, VAN, VN, SMN, LN, DGM) were extracted for pwMS and HC, including promiscuity, flexibility, cohesion, and disjointedness based on the Dynamic Graph Metrics toolbox (Sizemore and Bassett 2018). Promiscuity (Figure 1) quantifies how many networks a brain region was assigned to in relation to the total number of networks (number of networks switched to). Flexibility describes how many times a region was reassigned over time (total number of switches). Flexible switches can be further distinguished using cohesion and disjointedness. Cohesion quantifies how many of the reconfigurations are made together with another brain region (mutual switches) and disjointedness describes how many reconfigurations are made independently (independent switches) (Sizemore and Bassett 2018).

### 2.9 Statistical Analyses

All data were analyzed with the Statistical Package of Social Science (IBM SPSS Statistics 29). The level of significance was set to *p* < 0.05. Shapiro–Wilk test was applied to assess normal distribution of all variables, and we controlled for outliers.

*T*‐tests or Chi‐Square tests were performed to explore differences between pwMS and HC in demographics, neuropsychological, and structural/functional MRI data. Additionally, Pearson correlations were used to analyze associations between fatigue, demographics, and clinical data, calculated separately for pwMS and HC. Using ANCOVAs, differences in time‐varying reconfiguration parameters were analyzed while controlling for age, sex, and education, and *p*‐values were adjusted for multiple comparison using Bonferroni (adjusted *p*‐values are reported).

To investigate the association between fatigue and time‐varying brain network reconfigurations, we used a correlation‐based approach rather than dichotomizing participants into fatigued and non‐fatigued groups. Fatigue is a continuous variable, and splitting participants would result in a loss of detailed information and statistical power (Royston et al. 2006). Pearson correlations were computed to assess the association between all fatigue metrics and global as well as network‐specific reconfigurations, separately for pwMS and HC.

All correlations were Bonferroni‐corrected for multiple comparisons (adjusted *p*‐values reported).

To further investigate the relationship between fatigue and global reconfigurations, we performed hierarchical regression analyses (method: ENTER) only when significant correlations existed between fatigue (total, motor, and cognitive) and global reconfiguration parameters. Regression models controlled for demographics (age, sex, education), clinical (EDSS, DD, DMT), and structural MRI data (whole brain volume, T2‐LL). DMT was included as a categorical variable with the following categories: none, dimethyl fumarate, fingolimod, glatiramer, interferon, natalizumab, ocrelizumab, siponimod, and teriflunomid. Hierarchical regression analyses were performed only when significant correlations existed between fatigue (total, motor, or cognitive) and global reconfiguration parameters (promiscuity, flexibility, cohesion, or disjointedness).

In our sample, 15 pwMS were treated with interferons, which are known to potentially induce fatigue‐like symptoms and could therefore bias fatigue assessment. To evaluate this potential impact, we repeated all analyses excluding these patients and found that the results remained unchanged. Accordingly, the results reported in this manuscript are based on the full sample (*N* = 155).

A score > 11 was considered as a clinically significant depression/anxiety. In our sample, 7 pwMS met criteria for clinically significant depression and 22 for clinically significant anxiety. All analyses were repeated after excluding these participants, with unchanged results. Therefore, these participants were retained in the final analyses.

### 2.10 Validation Analyses

Recent work has emphasized the importance of incorporating additional validation steps in time‐varying analyses, particularly through the use of null models (Broeders et al. 2022). These models help determine whether observed effects result from random noise or static connectivity changes. We created a null distribution by phase‐randomizing the original time series following a Fourier transformation. Per participant, 50 randomization runs were performed, and the average was used as surrogate data. Then, the entire time‐varying pipeline was applied again using the generated surrogate data. Finally, ANOVAs evaluating the differences between pwMS and HC in global time‐varying reconfigurations (promiscuity, flexibility, cohesion, disjointedness) were analyzed again including the respective null model as a covariate (Broeders et al. 2022).

We additionally explored the effect of different window‐sizes by analyzing differences between pwMS and HC in global time‐varying reconfiguration parameters using a window‐size of 44s (Broeders et al. 2022).

The results of the validation analyses are reported in the Supporting Information (Supplement 1).

## 3 Results

### 3.1 Characteristics of pwMS and HC

Detailed information on demographics, clinical, neuropsychological, and structural MRI data is presented in Table 1.

**TABLE 1:** Patients' characteristics.

Demographics and clinical data	PwMS *N* = 155	HC *N* = 48	*p*
Age, mean (SD) (years)	39 (10)	33 (10)	**< 0.001** ^a^, ^b^
Sex (female), *N* (%)	96 (61.9)	34 (70.8)	0.262^c^
Education, mean (SD) (years)	14 (3)	17 (3)	**< 0.001** ^a^, ^b^
Disease duration, mean (SD) (years)	10 (8)	N.A.	
EDSS, median (IQR)	1.0 (2.0)	N.A.	
DMT, *N* (%)			
None	37 (23.9)	N.A.	
Dimethyl fumarate	52 (33.5)	N.A.	
Fingolimod	9 (5.8)	N.A.	
Glatiramer acetate	9 (5.8)	N.A.	
Interferon beta	15 (9.7)	N.A.	
Natalizumab	6 (3.9)	N.A.	
Ocrelizumab	13 (8.4)	N.A.	
Siponimod	8 (5.2)	N.A.	
Teriflunomid	6 (3.9)	N.A.	
Clinical phenotype, *N* (%)			
CIS	8 (5.2)	N.A.	
RRMS	135 (87.1)	N.A.	
SPMS	10 (6.5)	N.A.	
PPMS	2 (1.3)	N.A.	
Neuropsychological assessment			
Total fatigue, mean (SD)	50.3 (18.9)	35.8 (10.1)	**< 0.001** ^a^, ^b^
Motor fatigue, mean (SD)	25.9 (10.5)	17.3 (5.2)	**< 0.001** ^a^, ^b^
Cognitive fatigue, mean (SD)	24.4 (9.7)	18.5 (5.4)	**< 0.001** ^a^, ^b^
Depression, mean (SD)	3.7 (3.4)	1.7 (1.7)	**< 0.001** ^a^, ^b^
Clinically relevant depression, *N* (%)	7 (4.5)	0 (0)	
Anxiety, mean (SD)	5.7 (3.8)	3.8 (2.6)	**< 0.001** ^a^, ^b^
Clinically relevant anxiety, *N* (%)	22 (14.2)	0 (0)	
Brain MRI‐parameters			
T2‐LL, median (IQR) (cm^3^)	3.5 (0.7)	N.A.	
NBV, mean (SD) (cm^3^)	1512.1 (79.2)	1572.9 (58.7)	**< 0.001** ^a^, ^b^
Relative motion in rsfMRI, mean (SD) (mm)	0.14 (0.05)	0.13 (0.4)	**0.426** ^b^

*Note:* Bold value indicates *p* < 0.05.

Abbreviations: CIS, clinically isolated syndrome; DMT, disease modifying treatment; EDSS, Expanded Disability Status Scale; HC, healthy controls; IQR, inter quartile range; MRI, magnetic resonance imaging; *N*, number of participants; N.A., not available; NBV, normalized brain volume; PPMS, primary progressive multiple sclerosis; PwMS, people with multiple sclerosis; RRMS, relapse remitting multiple sclerosis; rsfMRI, resting‐state functional magnetic resonance imaging; SD, standard deviation; SPMS, secondary progressive multiple sclerosis; T2‐LL, T2‐lesion‐load.

^a^ Indicates *p* < 0.05; *N* = 155 pwMS; *N* = 48 HC.

^b^ Analyzed using a *t*‐test.

^c^ Analyzed using a Chi‐Square test.

Of pwMS, 89 (57.4%) had at least mild total fatigue (15 [9.7%] mild, 34 [21.9%] moderate, 40 [25.8%] severe total fatigue). More precisely, 93 (60%) pwMS had motor (23 [14.8%] mild, 22 [14.2%] moderate, 48 [31%] severe motor fatigue) and 89 (57.4%) pwMS had cognitive (28 [18.1%] mild, 32 [20.6%] moderate, 29 [18.7%] severe cognitive fatigue) fatigue.

Of HC, 10 (20.9%) had at least mild total fatigue (7 [14.6%] mild, 2 [4.2%] moderate, 1 [2.1%] severe total fatigue). More precisely, 8 (16.7%) HC had motor (5 [10.4%] mild, 3 [6.3%] moderate motor fatigue) and 14 (29.2%) pwMS had cognitive (12 [25%] mild, 1 [2.1%] moderate, 1 [2.1%] severe cognitive fatigue) fatigue.

PwMS had significantly higher total, motor, and cognitive fatigue scores than HC (all *p* < 0.001; Table 1). Total fatigue and motor fatigue were correlated with demographics and clinical data only in pwMS (Supplement 2).

### 3.2 Global Network Reconfiguration in pwMS and HC

No significant differences (Table 2) were observed between pwMS and HC in global promiscuity (pwMS: *M* = 0.35 ± 0.05, HC: *M* = 0.36 ± 0.05; *p* = 0.992), flexibility (pwMS: *M* = 0.25 ± 0.05, HC: *M* = 0.26 ± 0.04; *p* = 0.691), cohesion (pwMS: *M* = 0.17 ± 0.04, HC: *M* = 0.19 ± 0.04; *p* = 0.187), and disjointedness (pwMS: *M* = 0.07 ± 0.01, HC: *M* = 0.07 ± 0.01; *p* = 0.081).

**TABLE 2:** Differences between pwMS and HC in global time‐varying reconfiguration parameters.

Time‐varying reconfiguration parameters	PwMS *N* = 155	HC *N* = 48	*p*
Promiscuity, mean (SD)	0.36 (0.05)	0.35 (0.05)	0.992^a^
Flexibility, mean (SD)	0.26 (0.04)	0.25 (0.04)	0.691^a^
Cohesion, mean (SD)	0.19 (0.04)	0.17 (0.04)	0.187^a^
Disjointedness, mean (SD)	0.07 (0.01)	0.07 (0.01)	0.081^a^

Abbreviations: HC, healthy controls; *N*, number of participants; PwMS, people with multiple sclerosis; SD, standard deviation.

^a^ Analyzed using an ANCOVA, corrected for age, sex, education and multiple comparisons (Bonferroni).

### 3.3 Global Network Reconfigurations Associated With Fatigue

#### 3.3.1 Total Fatigue in pwMS

In pwMS, higher total fatigue was significantly correlated with greater global promiscuity (*r* = 0.21, *p* = 0.032) and disjointedness (*r* = 0.24, *p* = 0.008; Table 3).

**TABLE 3:** Pearson correlations between global time‐varying reconfigurations and fatigue in pwMS and HC.

Time‐varying global reconfigurations	Total fatigue				Motor fatigue				Cognitive fatigue			
	PwMS, *N* = 155		HC, *N* = 48		PwMS, *N* = 155		HC, *N* = 48		PwMS, *N* = 155		HC, *N* = 48	
	*r*	*p*	*r*	*p*	*r*	*p*	*r*	*p*	*r*	*p*	*r*	*p*
Promiscuity	**0.21**	**0.032** ^a^	−0.03	0.844	**0.25**	**0.008** ^a^	0.01	0.969	0.14	0.292	−0.06	0.684
Flexibility	0.18	0.096	−0.01	0.986	**0.21**	**0.032** ^a^	0.04	0.773	0.12	0.524	−0.05	0.757
Cohesion	0.14	0.324	0.04	0.793	0.17	0.148	0.04	0.688	0.09	0.255	0.03	0.829
Disjointedness	**0.24**	**0.008** ^a^	0.04	0.798	**0.28**	**< 0.001** ^a^	0.06	0.784	0.18	0.124	0.02	0.916

*Note:* Bold value indicates significant using *p* < 0.05.

Abbreviations: HC, healthy controls; *p*, *p*‐value corrected for multiple comparisons (Bonferroni); PwMS, people with multiple sclerosis; *r*, Pearson correlation coefficient.

^a^ Indicates significant using *p* < 0.05.

In the hierarchical regression model, total fatigue (adj.*R* ^2^ = 0.23, *p* < 0.001) remained associated with global disjointedness (*β* = 0.17, *p* = 0.033) after controlling for demographics (age, sex, education), clinical data (EDSS, DD, DMT), and structural brain damage (whole brain volume, T2‐LL).

#### 3.3.2 Motor Fatigue in pwMS

Motor fatigue was similarly correlated with higher global promiscuity (*r* = 0.25, *p* = 0.008), flexibility (*r* = 0.21, *p* = 0.032), and disjointedness (*r* = 0.28, *p* < 0.001; Table 3).

This association (adj.*R* ^2^ = 0.38, *p* < 0.001) remained significant in the hierarchical regression model for global disjointedness (*β* = 0.16, *p* = 0.026) after adjusting for demographics (age, sex, education), clinical data (EDSS, DD, DMT), and structural brain damage (whole brain volume, T2‐LL).

#### 3.3.3 Cognitive Fatigue in pwMS

In contrast, cognitive fatigue showed no significant association with any global network reconfiguration parameter (Table 3).

#### 3.3.4 Fatigue in HC

No significant correlations were found between fatigue and global network reconfigurations in HC (all *p* > 0.05; Table 3).

### 3.4 Network‐Specific Reconfigurations Associated With Fatigue

#### 3.4.1 Total Fatigue in pwMS

After Bonferroni correction, we observed a significant correlation between total fatigue and promiscuity in the limbic network (*r* = 0.25, *p* = 0.016).

#### 3.4.2 Motor Fatigue in pwMS

Similar to total fatigue, higher motor fatigue was correlated with network promiscuity in the limbic network (*r* = 0.25, *p* = 0.016).

#### 3.4.3 Cognitive Fatigue in pwMS

We did not observe any significant correlations between cognitive fatigue and network‐specific reconfigurations in pwMS (all *p* > 0.05).

#### 3.4.4 Fatigue in HC

No significant correlations between fatigue and network‐specific reconfigurations were observed in HC (all *p* > 0.05).

## 4 Discussion

In this cross‐sectional study, we examined time‐varying global and network‐specific reconfigurations, specifically promiscuity, flexibility, cohesion, and disjointedness, in pwMS and HC in relation to total, motor, and cognitive fatigue. Our results showed that higher total and motor fatigue in pwMS is linked to more extensive reconfigurations in terms of both global time‐varying brain network and network‐specific reconfigurations in the limbic network (LN). Notably, these associations were primarily driven by motor fatigue, reflecting the fact that total fatigue comprises both motor and cognitive components. These associations were not present in our HC sample, which may reflect differences in the dynamics of brain network reconfigurations related to fatigue between pwMS and healthy individuals. While this suggests a potential role of network reconfigurations in MS‐related fatigue, further studies are needed to confirm these findings.

### 4.1 Associations Between Fatigue and Global Time‐Varying Network Reconfigurations in pwMS

Higher total and motor fatigue were associated with greater promiscuity, flexibility, and disjointedness in pwMS. Notably, the link with disjointedness, reflecting more independent switches of brain regions, remained significant even after adjusting for demographics, clinical data, and structural brain damage. This suggests that MS‐related fatigue is associated with a more unstable functional brain network.

These findings align with earlier studies using different time‐varying metrics (e.g., global time‐varying functional connectivity) in smaller samples to explore time‐varying functional connectivity changes to MS‐related fatigue in the DMN (Romanello et al. 2022; Tijhuis et al. 2021). However, it remains an open question whether changes in the time‐varying functional brain networks lead to greater fatigue or alternatively if fatigue represents a state of reduced availability of energy which may influence regulatory self‐control of the network.

On the one hand, our results can be understood by the hypothesis that changes in time‐varying functional brain networks are involved in the development of perceived fatigue in MS. Multiple factors have been implicated in the pathogenesis of MS‐related fatigue, including inflammation, brain damage, and metacognitive mechanisms (Manjaly et al. 2019). Yet, a review suggested that fatigue in MS primarily results from functional alterations in the communication of involved networks rather than from structural brain changes (Bertoli and Tecchio 2020). MS‐related structural damage of white matter and grey matter, along with inflammatory processes within and outside the central nervous system, might lead to maladaptive network recruitment (Manjaly et al. 2019). This atypical network reorganization could subsequently result in the (compensatory) recruitment of additional brain regions not usually involved in a given task and/or increased activation within the network, ultimately leading to higher perceived fatigue due to enhanced load on the network (Manjaly et al. 2019; Chalah and Ayache 2024). Furthermore, a recent study showed that more increased time‐varying functional reconfigurations require more energy which might also lead to more subjectively perceived fatigue (Broeders, Van Dam, et al. 2024). This interpretation aligns with our findings, which demonstrate greater instability in brain networks associated with increased fatigue. Another potential mechanism through which functional network disturbances may lead to fatigue is that more unstable brain networks could impair the brain's self‐monitoring mechanisms, supporting the metacognitive theory (Manjaly et al. 2019; Stephan et al. 2016). According to this theory, fatigue may arise from the brain's inference about its capacity for control (Manjaly et al. 2019), which could be influenced by the instability of brain networks.

On the other hand, our results could also be interpreted differently: as fatigue increases, the brain may lose more of its self‐regulatory control (Manjaly et al. 2019), leading to greater instability in brain networks. However, it remains unclear whether this potential increase in instability serves as a compensatory process. Structural brain damage is known to heighten the effort required for compensatory mechanisms, which contributes to the pathogenesis of fatigue in MS (Capone et al. 2020). This process may create a vicious cycle: compensatory mechanisms require greater effort, leading to higher perceived fatigue, which in turn necessitates further compensatory processes.

### 4.2 Associations Between Fatigue and Time‐Varying Network Reconfigurations in pwMS

In addition to global time‐varying reconfigurations, we found that higher total and motor fatigue were associated with increased promiscuity in the limbic network.

Recent studies reported associations between fatigue and the (meso‐) limbic system in pwMS (Damasceno et al. 2016; Palotai et al. 2019; Pardini et al. 2010; Carandini et al. 2021). On a structural level, earlier research identified grey matter atrophy in the (meso‐) limbic system (Damasceno et al. 2016; Palotai et al. 2019) and damage to white matter tracts in fronto‐limbic pathways in pwMS with fatigue (Pardini et al. 2010). The mesocorticolimbic system, particularly its dopaminergic pathways to the nucleus accumbens and other limbic structures forming the brain's reward system, appears to be strongly associated with fatigue in MS (Carandini et al. 2021). Furthermore, altered static resting‐state functional connectivity in LN regions has also been associated with MS‐related fatigue, as reported in a recent review by Kampaite et al. (2024) (Kampaite et al. 2024). Our findings add to this by highlighting that time‐varying aspects of LN reconfiguration may also contribute to fatigue severity.

### 4.3 Differences in Time‐Varying Reconfigurations Between pwMS and HC

In contrast to a previous study (von Schwanenflug et al. 2023), that reported alterations in time‐varying reconfigurations between pwMS and HC, we did not find significant differences between these groups in the present study. One possible explanation is that, compared with the above mentioned study (von Schwanenflug et al. 2023), our MS cohort was only mildly impaired, with a median EDSS of 1.0 and only about half of the pwMS experiencing at least mild fatigue. Furthermore, the majority of pwMS had RRMS as their clinical phenotype and were receiving DMT. These factors may account for the absence of significant differences in time‐varying reconfigurations between pwMS and HC in our sample. Notably, another study (Broeders et al. 2022) similarly did not find general differences in time‐varying reconfigurations between pwMS and HC.

Although 20% of HC reported at least mild fatigue, we did not observe any associations between fatigue and time‐varying reconfigurations in this cohort. In particular, only three HC reported moderate or severe fatigue. Therefore, fatigue in our HC sample might be more likely influenced by lifestyle and psychological factors rather than changes in brain networks (Finsterer and Mahjoub 2014). Furthermore, the association between fatigue and time‐varying reconfigurations may be unique to MS‐related fatigue.

### 4.4 No Associations Between Cognitive Fatigue and Network Reconfigurations

Interestingly, in contrast to motor fatigue, cognitive fatigue was not associated with any global or network reconfiguration measures in our cohort. This aligns with the notion that the underlying brain mechanisms differ between cognitive and motor fatigue, as we also observed in an earlier study from our group (Hechenberger et al. 2023).

Previous research has linked higher cognitive fatigue in pwMS to both structural (lower fractional anisotropy in the cortico‐striato‐thalamo‐cortical loop (Hechenberger et al. 2023)) as well as functional (e.g., lower functional connectivity between the insula and cingulate, the thalamus and operculum (Stefancin et al. 2019)) brain alterations. Additionally, decreased noradrenaline transporter–enriched connectivity in several frontal and prefrontal regions has been linked to greater cognitive fatigue in pwMS (Cercignani et al. 2021).

Taken together, our findings underline the importance of investigating distinct contributors to motor and cognitive fatigue, given that some pwMS are primarily affected by cognitive fatigue (Hechenberger et al. 2023) and the underlying mechanism may differ.

## 5 Limitations

The current study provides valuable insights into the association between MS‐related fatigue and time‐varying reconfigurations. However, several limitations must be considered. First, we primarily included pwMS with RRMS and only a few with progressive MS, which limits the generalizability of our findings across the entire MS spectrum. People with progressive MS are often excluded from clinical studies due to practical challenges related to including pwMS with a worse disease phenotype (Lechner‐Scott et al. 2022), as well as MRI processing related complications (Filippi et al. 2021). Additional studies need to establish whether our findings can be replicated in progressive MS.

Second, due to our cross‐sectional design, we could not determine whether fatigue results from a more unstable brain network or if increased flexibility serves as a compensatory mechanism. Therefore, evaluating these findings in a longitudinal study would be of great interest, preferably early in the disease so we can evaluate how the development of fatigue relates to time‐varying reconfiguration parameters.

Third, compared with our pwMS cohort our HC group was relatively small. A larger HC sample would allow more robust comparisons of correlations between fatigue scores and time‐varying reconfigurations. However, fatigue in healthy populations is typically low and strongly skewed. Only 10% to 18% of the general population report chronic fatigue without meeting the criteria for chronic fatigue syndrome (David et al. 1990; Pawlikowska et al. 1994; Yoon et al. 2023). Even with a larger sample, the distribution of fatigue scores in HC would likely remain limited.

A further limitation of this study is the relatively short duration of the resting‐state fMRI acquisition. Recent work suggests that longer resting‐state scans (e.g., 20–30 min) provide more stable estimates of functional and time‐varying connectivity, while scan durations of ~10 min can be considered moderately stable (Ma et al. 2024; Ooi et al. 2025). Due to feasibility constraints typical of clinical MS research settings, the resting‐state acquisition in the present study was shorter. Although meaningful estimates of time‐varying connectivity can still be obtained from such data, future studies employing longer resting‐state acquisitions will be important to confirm and extend the current findings.

Another limitation of the present study is the absence of cerebellar regions of interest. Neither the Brainnetome atlas nor the FIRST‐based segmentation includes cerebellar parcellations, and in addition, cerebellar data were affected by artifacts in some participants, further precluding reliable assessment of cerebellar network dynamics. This is relevant given emerging evidence linking cerebellar network properties, including centrality measures, to fatigue in MS (Hok et al. 2025). Future studies incorporating dedicated cerebellar parcellations and improved acquisition methods will be important to capture cerebellar contributions to time‐varying network reconfigurations and provide a more comprehensive understanding of MS‐related fatigue.

Furthermore, we did not apply a specific quality control pipeline (e.g., using the CONN toolbox) (Morfini et al. 2023). However, motion was monitored using parameters from the FEAT reports (MCFLIRT), and participants with excessive motion would have been excluded. In addition, we extracted mean relative motion from the FEAT reports and tested for group differences between pwMS and HC. Since we did not find any differences, it is unlikely that motion has driven group differences or spurious correlations.

Another limitation is that the limbic network comprises regions prone to susceptibility‐related signal loss. Although regions with insufficient voxel coverage were excluded and participant‐wise voxel coverage estimates were used as proxies for signal reliability. These estimates did not differ significantly between pwMS and HC (all *p* > 0.05). Nevertheless, voxel coverage does not fully capture signal quality (e.g., temporal signal‐to‐noise ratio), so residual effects of signal loss on time‐varying connectivity and network reconfigurations in this area cannot be entirely excluded.

Lastly, structural and functional disease‐related brain damage are likely to jointly contribute to the development of fatigue (Manjaly et al. 2019). Hence, future studies should aim to understand the unique contribution of time‐varying reconfiguration parameters as well as further investigate how structural and functional changes influence each other in the context of fatigue in pwMS.

## 6 Conclusion

This work identifies time‐varying brain network instability as a functional correlate of fatigue in MS, independent of structural brain damage. More unstable reconfigurations may increase the cognitive and physical effort required to maintain network effectiveness, or may represent compensatory processes that themselves contribute to exhaustion. In particular, time‐varying alterations of the limbic network highlight the potential role of motivational and reward circuits in sustaining fatigue.

These findings advance mechanistic understanding of MS‐related fatigue and suggest that time‐varying connectivity measures could complement structural and static functional markers in future studies. Next, we need to understand which pathological processes lead to network instability. Furthermore, longitudinal investigations will be critical to determine whether network instability predicts the onset or worsening of fatigue, and, thereby, whether it can serve as a marker to monitor disease progression or treatment response for pwMS.

## Author Contributions

Stefanie Hechenberger: Conceptualization, Methodology, Formal analysis, Writing – Original Draft, Visualization; Tommy A.A. Broeders: Methodology, Writing – Review and Editing; Marloes D.A. Bet: Writing – Review and Editing; Birgit Helmlinger: Writing – Review and Editing; Christian Tinauer: Methodology, Writing – Review and Editing; Stefan Ropele: Data Curation, Writing – Review and Editing; Sebastian Wurth: Writing – Review and Editing; Anna Damulina: Writing – Review and Editing; Michael Khalil: Writing – Review and Editing; Menno M. Schoonheim: Supervision, Writing – Review and Editing; Christian Enzinger: Resources, Writing – Review and Editing; Daniela Pinter: Conceptualization, Validation, Supervision, Writing – Review and Editing.

## Ethics Statement

This project was approved by the Ethics Committee of the Medical University of Graz (31–432 ex 18/191264–2019).

## Consent

All participants included in the study gave written informed consent.

## Conflicts of Interest

The author(s) declared the following potential conflicts of interest with respect to the research, authorship, and/or publication of this article: S.H. has received speaking honoraria from Roche, Bristol‐Myers Squibb, and Merck. T.A.A.B. receives research support from the Dutch MS Research Foundation. M.D.A.B. has nothing to disclose. B.Hel. has received funding for travel from Janssen and speaking honoraria from Roche, Bristol‐Myers Squibb, and Sanofi. C.T. has no disclosures. S.R. has no disclosures. B.Hes. has received funding for travel or speaker honoraria from Bayer, Biogen, Bristol‐Myers Squibb, Janssen, Merck, Novartis, Roche, Sanofi‐Genzyme and Teva. S.W. has participated in meetings sponsored by, received honoraria or travel funding from Allergan, Biogen, Ipsen Pharma, Merck, Novartis, Roche, Sanofi Genzyme, Teva and Bristol Myers Squibb. A.D. has participated in meetings sponsored by, received speaker honoraria or travel funding from Sanofi‐Aventis, Novartis and Janssen. M.K. has received travel funding and speaker honoraria from Bayer, Biogen, Novartis, Merck, Sanofi and Teva and serves on scientific advisory boards for Biogen, Bristol‐Myers Squibb, Gilead, Merck, Neuraxpharm, Novartis, Alexion, Amgen and Roche. He received research grants from Biogen, Novartis and Teva. M.M.S. serves on the editorial board of Neurology and Frontiers in Neurology, receives research support from the Dutch MS Research Foundation, Eurostars‐EUREKA, ARSEP, Amsterdam Neuroscience, MAGNIMS and ZonMW and has served as a consultant for or received research support from Atara Biotherapeutics, Biogen, Celgene/Bristol Meyers Squibb, EIP, Sanofi, MedDay and Merck. C.E. received funding for traveling and speaker honoraria from Biogen Idec, Bayer Schering Pharma, Merck Serono, Novartis, Genzyme and Teva Pharmaceutical Industries Ltd./sanofi‐aventis, Shire; received research support from Merck Serono, Biogen Idec, and Teva Pharmaceutical Industries Ltd./sanofi‐aventis; and serves on scientific advisory boards for Bayer Schering Pharma, Biogen Idec, Merck Serono, Novartis, Genzyme, Roche, and Teva Pharmaceutical Industries Ltd./sanofi Aventis. D.P. is a member of the advisory board for “Cognition and MS” for Novartis and has received speaking honoraria from Biogen, Novartis, MedAhead, and Bristol‐Myers Squibb.

## Supporting information


**Data S1:** Supporting Information.

## Data Availability

The data that support the findings of this study are available on request from the corresponding author. The data are not publicly available due to privacy or ethical restrictions.
