# Supplementary material for: Time‐Varying Brain Functional Reconfiguration Patterns Associated With Fatigue in Multiple Sclerosis
Source: Hum Brain Mapp. 2026 Mar 8;47(4):e70480. doi: 10.1002/hbm.70480 (PMC12968462; doi:10.1002/hbm.70480)
Supplement: Supplementary file 1 — Data S1: Supporting Information. [file HBM-47-e70480-s001.docx]

**Supplement 1. Results of the validation analyses**

The results regarding group differences between pwMS and HC in global promiscuity (pwMS: *M*=0.36±0.05, HC: *M*=0.35±0.05; *p*=0.764), flexibility (pwMS: *M*=0.26±0.04, HC: *M*=0.25±0.04; *p*=0.514), cohesion (pwMS: *M*=0.19±0.04, HC: *M*=0.17±0.04; *p*=0.201), and disjointedness (pwMS: *M*=0.07±0.01, HC: *M*=0.07±0.01; *p*=0.295) remained unchanged when incorporating the respective null model into the ANOVAs.

When controlling for age, sex, and education, similar results were obtained using a shorter window size of 44 seconds, with no significant group differences in promiscuity (pwMS: *M*=0.46±0.06, HC: *M*=0.46±0.05; *p*=0.293), flexibility (pwMS: *M*=0.33±0.04, HC: *M*=0.32±0.04; *p*=0.652), cohesion (pwMS: *M*=0.25±0.04, HC: *M*=0.25±0.04; *p*=0.856), or disjointedness (pwMS: *M*=0.08±0.01, HC: *M*=0.08±0.01; *p*=0.051).

For all analyses the initial parameter settings were retained.

**Supplement 2. Correlation between fatigue, demographics, and clinical data in pwMS and HC**

|  | **Total fatigue** | | | | **Motor fatigue** | | | | **Cognitive fatigue** | | | |
| --- | --- | --- | --- | --- | --- | --- | --- | --- | --- | --- | --- | --- |
|  | PwMS, *N*=155 | | HC, *N*=48 | | PwMS, *N*=155 | | HC, *N*=48 | | PwMS, *N*=155 | | HC, *N*=48 | |
|  | *r* | *p* | *r* | *p* | *r* | *p* | *r* | *p* | *r* | *p* | *r* | *p* |
| **Demographics** |  |  |  |  |  |  |  |  |  |  |  |  |
| Age | 0.15 | 0.072 | -0.26 | 0.081 | **0.24** | **0.009*** | 0.25 | 0.090 | 0.02 | 0.784 | 0.17 | 0.246 |
| Sex | -0.09 | 0.286 | 0.22 | 0.135 | -0.06 | 0.431 | -0.22 | 0.131 | -0.10 | 0.222 | -0.26 | 0.071 |
| Education | -0.18 | 0.066 | -0.02 | 0.874 | **-0.20** | **0.045*** | -0.05 | 0.734 | -0.15 | 0.068 | 0.01 | 0.975 |
| **Clinical data** |  |  |  |  |  |  |  |  |  |  |  |  |
| Disease duration | 0.15 | 0.073 | N.A. | N.A. | **0.23** | **0.015*** | N.A. | N.A. | 0.04 | 0.645 | N.A. | N.A. |
| EDSS | **0.42** | **0.003*** | N.A. | N.A. | **0.58** | **<0.001*** | N.A. | N.A. | 0.19 | 0.063 | N.A. | N.A. |
| DMT | 0.10 | 0.208 | N.A. | N.A. | 0.09 | 0.267 | N.A. | N.A. | 0.10 | 0.212 | N.A. | N.A. |
| **Brain MRI-parameters** |  |  |  |  |  |  |  |  |  |  |  |  |
| T2-LL | **0.25** | **0.002*** | N.A. | N.A. | **0.26** | **0.001*** | N.A. | N.A. | **0.21** | **0.008*** | N.A. | N.A. |
| NBV | -0.14 | 0.079 | 0.05 | 0.758 | **-0.20** | **0.011*** | 0.01 | 0.952 | -0.06 | 0.493 | 0.08 | 0.603 |

PwMS: people with multiple sclerosis; HC: healthy controls; *r*: Pearson correlation coefficient; *p*: p-value corrected for multiple comparisons (Bonferroni); N.A.: not available; EDSS: Expanded Disability Status Scale; DMT: disease modifying treatment; MRI: magnetic resonance imaging; T2-LL: T2-lesion-load; NBV: normalized brain volume

* indicates significant using p < 0.05
